# Three-dimensional computer graphic animations for studying social approach behaviour in medaka fish: Effects of systematic manipulation of morphological and motion cues

**DOI:** 10.1371/journal.pone.0175059

**Published:** 2017-04-11

**Authors:** Tomohiro Nakayasu, Masaki Yasugi, Soma Shiraishi, Seiichi Uchida, Eiji Watanabe

**Affiliations:** 1 Laboratory of Neurophysiology, National Institute for Basic Biology, 5–1 Higashiyama, Myodaiji-cho, Okazaki, Aichi, Japan; 2 Department of Molecular & Cellular Physiology, Shinshu University School of Medicine, 3-1-1 Asahi, Matsumoto, Nagano, Japan; 3 Human Interface Laboratory, Department of Advanced Information Technology, Faculty of Information Science and Electrical Engineering, Kyushu University, Motooka, Nishi-ku, Fukuoka, Japan; 4 NEC Corporation, 5-7-1, Shiba, Minato-ku, Tokyo, Japan; 5 Department of Basic Biology, Faculty of Life Science, SOKENDAI (Graduate University for Advanced Studies), 5–1 Higashiyama, Myodaiji-cho, Okazaki, Aichi, Japan; Rijksuniversiteit Groningen, NETHERLANDS

## Abstract

We studied social approach behaviour in medaka fish using three-dimensional computer graphic (3DCG) animations based on the morphological features and motion characteristics obtained from real fish. This is the first study which used 3DCG animations and examined the relative effects of morphological and motion cues on social approach behaviour in medaka. Various visual stimuli, e.g., lack of motion, lack of colour, alternation in shape, lack of locomotion, lack of body motion, and normal virtual fish in which all four features (colour, shape, locomotion, and body motion) were reconstructed, were created and presented to fish using a computer display. Medaka fish presented with normal virtual fish spent a long time in proximity to the display, whereas time spent near the display was decreased in other groups when compared with normal virtual medaka group. The results suggested that the naturalness of visual cues contributes to the induction of social approach behaviour. Differential effects between body motion and locomotion were also detected. 3DCG animations can be a useful tool to study the mechanisms of visual processing and social behaviour in medaka.

## Introduction

Many species of fish form and maintain social aggregations called shoals [1]. It appears that shoals have several functions, including reduction in predation risk [2, 3], enhancement of feeding [4, 5], mating opportunities [6], and hydrodynamic advantages during locomotion [7]. The formative mechanism of fish shoals has been discussed in previous studies, and it appears to be based on individual perceptions and decisions, rather than on the steering by a fixed leader [8].

In the present study, we examined the visual cues that are critical for the induction of social approach behaviour, i.e., shoaling behaviour, in medaka fish (*Oryzias latipes*) using 3DCG. Medaka is a small freshwater teleost, which is a native of the Far East [9], and has been widely used in genetic and developmental studies [10]. The medaka brain atlas has also been published [11], and medaka can be a suitable experimental model to study the genetic and brain mechanisms underlying behaviour of aquatic organisms. Indeed, recent studies have revealed that visual contact of a mating partner (visual familiarization) enhanced the female preference for males and the neural mechanisms underlying the mating partner preference based on the visual familiarization [12] and that males remained a mating partner and repelled their rivals (mate-guarding behaviour) and the underlying neural systems which mediate mate-guarding behaviour [13], though these studies were done in the context of mating behaviour, not in the context of social approach behaviour. Although zebrafish model has quickly and widely spread, research using another fish model will complement and expand the previous results using zebrafish.

Many researchers have analysed shoaling and schooling-like behaviours in medaka fish [14–21], and medaka were dependent on visual cues to maintain proximity to conspecifics [15, 21] or mirror images of their own selves [17]. However, the detailed nature of visual attraction in medaka remains unclear. In another fish species, several visual features, such as shoal size [22–25], sex [24, 25], familiarity [22], body colouration [26, 27], and body size [22, 28, 29], affect the formation of fish shoals. Although previous studies have primarily focused on what role morphological features of visual cues have in the social approach behaviour, perception of visual motion is one of the most basic abilities of fish as well as other vertebrates [30, 31]. We recently showed that visual motion cues play critical roles in the decision making of medaka in studies on hunting behaviour against the modelled motion of zooplanktons [32] and social approach behaviour induced by biological motion stimuli of conspecific fish [33]. Refined modifications of motion cues have largely effects on social approach behaviour in medaka [33]. Various types of spatial and temporal features of visual cues can affect the formation of shoals of medaka in a varying degrees. However, what the relative effects of these features are on the formation of shoals remains unclear.

Previous studies have employed several experimental techniques, including live stimuli, mirrors, computer still images, and videotaped images to reveal the features which induce shoaling behaviour. Each technique has its own advantages, but researchers have found that it is difficult to systematically control and manipulate various features of complex stimuli. In principle it is impossible to control behaviour of live fish. Motion information is not associated with computer still images. The use of video allows us to edit basic motion information, including reverse playback, speed change, and frame rate reduction. However, this technique does not allow for more advanced manipulation of motion. For example, we cannot create fish on display in which the moving trajectory is exactly the same as real fish (i.e., locomotion information remains intact) but their tail fins do not move (i.e., lack of body motion). Virtual reality technology, including computer graphics (especially 3D modelling and animation), can overcome these problems of previous studies and enables us to examine which and how cues are important for inducing shoaling behaviour [34]. Although the presentation of two-dimensional (2D) animations could be sufficient to induce shoaling behaviour in zebrafish [35], 3DCG can reconstruct animal motion patterns more accurately than 2DCG and therefore motion patterns in 3D may appear more realistic to subject animals than with only two dimensions. However, regardless of the promising advantages, there are few behavioural studies using 3DCG animations with advanced manipulation of motion information (e.g., locomotion vs. body motion) in parallel with morphological information, besides other behavioural context using lizards [36] and chimpanzees [37].

In the current study, we constructed realistic 3DCG animations of medaka fish based on the morphological features and tracking coordinate data obtained from real medaka fish and systematically manipulated the morphological and motion visual cues to investigate their relative contributions to social approach behaviour. This study showed that although any manipulation of the visual cues reduced the time spent in social approach behaviour, the colour, shape, and locomotion were particularly important determinants of the induction of social approach behaviour. 3DCG animations will help shed light on studies on social behaviours of fish in future.

## Results

### Presentation of realistic 3DCG animations of medaka fish

**Fig 1:**
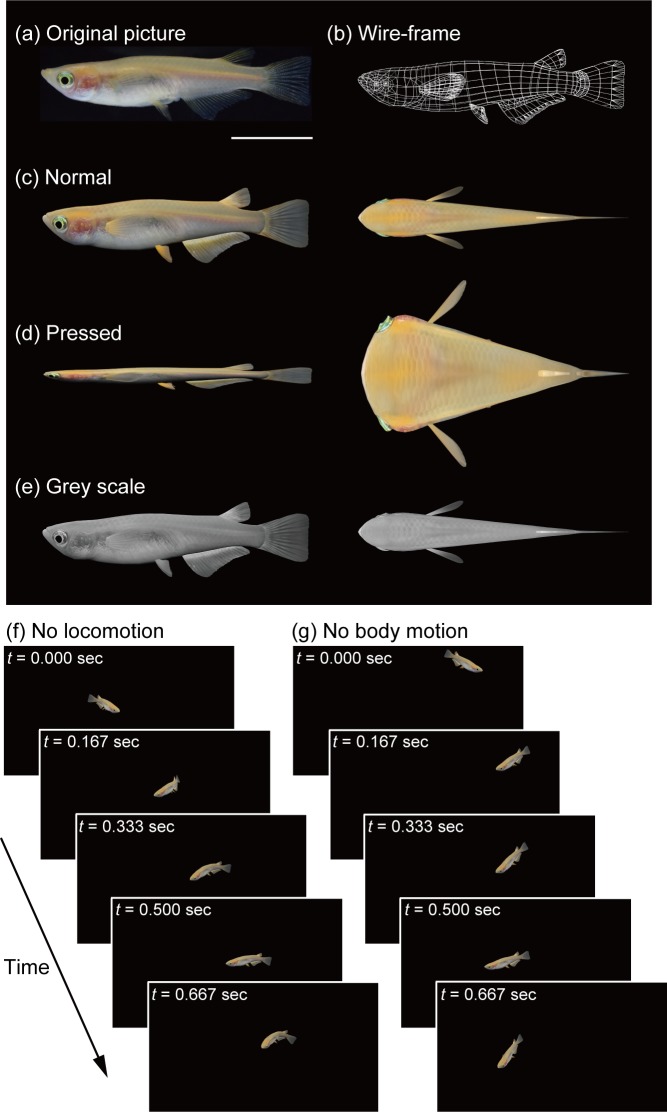
3D medaka models. 3DCG animations were created using pictures of real medaka fish (a). Scale bar indicates 1 cm. A wire-frame model of medaka was shown in b. Morphological and motion features were reproduced based on the medaka pictures and the tracking coordinate date (Normal (c)). We systematically modified the normal medaka model to generate altered animations in which shape (Pressed (d)), colour (Grey scale (e)), and motion (No locomotion (f) and No body motion (g)) were manipulated. The left and right panels in the normal, pressed, and grey scale medaka images show side and top views, respectively. In the No locomotion group, medaka fish were presented with 3DCG animations in which, although the motion of the tail fin of virtual medaka was intact, their locomotion was stopped. In the No body motion group, while the moving trajectory of virtual medaka was intact, their body axis motion was removed.

3DCG animations were presented to the subject medaka fish on a liquid crystal display (LCD). Based on high resolution photographs, 3D polygon models of female medaka fish were created using 3DCG software, including 3ds Max (Autodesk, CA) and Blender (https://www.blender.org/). To reconstruct the motion of medaka, we created a BioVision Hierarchy (BVH) file on the basis of the tracking coordinate data obtained from real fish. BVH files include skeleton hierarchy information and the motions of skeletons in each frame. We connected morphological and motion information using Blender. In the current study, social approach behaviour was assessed through the analysis of the time during which medaka were close to the display. Table 1 shows the experimental groups used in each experiment. We modified the colour, shape, locomotion, and body motion (Fig 1). Colour images were changed to grey scale (Grey scale group). The width of the body was tripled, and the height of the body was reduced to one-third of its original size (Pressed group). We stopped virtual fish locomotion, but recreated the motion of their tail fins (No locomotion group). In addition, we removed such body axis motion of the virtual fish, which resulted in a lack of body motion (No body motion group). The reason we chose females was because, in zebrafish, although males were preferred less by other males, both sexes were attracted to females [24].

**Table 1:** Experimental groups used in experiments 1 to 3.

	Visual characteristics			
	Form	Colour	Locomotion	Body motion
Experiment 1 (each *n* = 18)				
Normal	✓	✓	✓	✓
Static	✓	✓	×	×
Grey scale/Static	✓	×	×	×
Pressed/Static	×	✓	×	×
Pressed/Grey scale/Static	×	×	×	×
Blank	―	―	―	―
Experiment 2 (each *n* = 24)				
Normal	✓	✓	✓	✓
Grey scale/No locomotion	✓	×	×	✓
Grey scale/No body motion	✓	×	✓	×
Pressed/Grey scale	×	×	✓	✓
Pressed/No locomotion	×	✓	×	✓
Pressed/No body motion	×	✓	✓	×
Experiment 3 (each *n* = 24)				
Normal	✓	✓	✓	✓
Pressed	×	✓	✓	✓
Grey scale	✓	×	✓	✓
No locomotion	✓	✓	×	✓
No body motion	✓	✓	✓	×

In experiment 1, we presented experimental medaka fish with normal virtual medaka (see S1 Movie), several static images of virtual medaka, and no visual stimuli (Blank group). In experiment 2, two features of virtual medaka were simultaneously manipulated (for example, Grey scale/No locomotion and Pressed/No body motion groups, see S2 Movie). In experiment 3, we manipulated only one feature of the virtual medaka (see S3 Movie), which resulted in four experimental groups: Pressed, Grey scale, No locomotion, and No body motion.

After the baseline period (1 min), in which no stimuli were presented, visual stimuli were presented on the LCD screen (5 min). A one-way analysis of variance (ANOVA) revealed that during the baseline period, the time during which the fish were close to the display did not differ between groups (*p* > 0.05).

### Effects of visual motion on social approach behaviours

**Fig 2:**
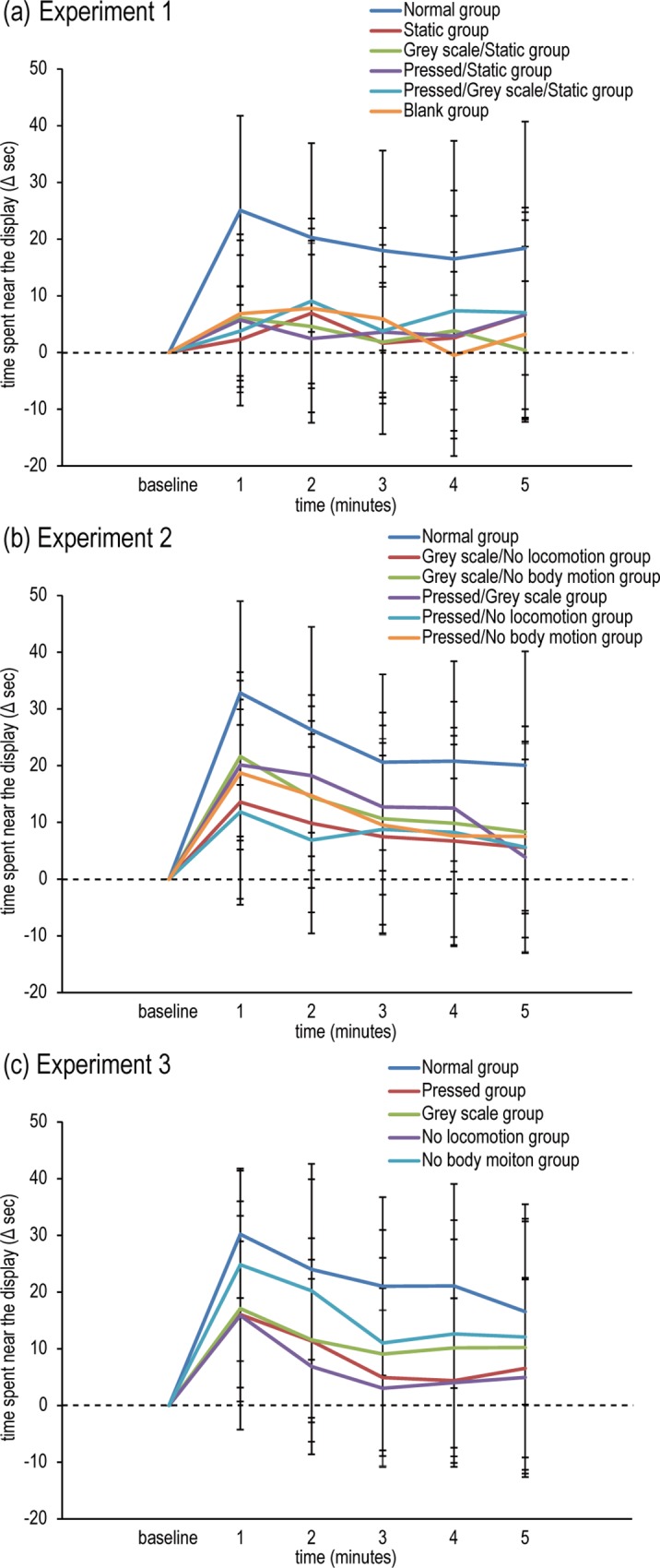
The results of experiments 1 to 3. In experiment 1 (a), the effects of normal virtual medaka were compared with those of several static images of virtual medaka (Static, Grey scale/Static, Pressed/Static, and Pressed/Grey scale/Static groups) and blank (Blank group). In experiment 2 (b), two features of normal virtual medaka were changed, which resulted in the following five experimental groups: Grey scale/No locomotion, Grey scale/No body motion, Pressed/Grey scale, Pressed/No locomotion, and Pressed/No body motion. In experiment 3 (c), a single feature of normal virtual medaka was changed, which resulted in the following four experimental groups: Pressed, Grey scale, No locomotion, and No body motion. We analysed the time during which medaka were close to the display. All data are expressed as the means ± SD.

In experiment 1, we found that the medaka reacted to normal virtual medaka to a considerable extent, but they showed little reaction to static images of virtual medaka. In experiment 1 (Fig 2A), medaka that were presented normal virtual medaka spent a long time in proximity to the display (within 10 mm from the display). On the other hand, the presentation of several types of static visual stimuli had negligible effects on the time spent near the display. Mendoza’s multisample sphericity test revealed that the sphericity assumption was not satisfied (*p* < 0.001). Therefore, the degrees of freedom were corrected using the Huynh-Feldt correction. A two-way ANOVA revealed significant main effects of group (*F*(5,102) = 5.33, *p* < 0.001) and time (*F*(3.57,363.87) = 7.72, *p* < 0.001). *Post-hoc* multiple comparisons using Ryan’s method showed that the Normal group spent significantly more time near the display than the other groups (*p* < 0.05) and that the time spent near the display in the stimulus presentation period was significantly increased when compared with the baseline period (*p* < 0.05). These results indicated that although the presentation of visual stimuli, *per se*, had effects on the induction of social approach behaviour, an increase in social approach behaviour was highly evident in the Normal group in which all four features (colour, shape, locomotion, and body motion) were reconstructed.

### Effects of changing two visual features on social approach behaviours

In experiment 2, it was shown that social approach behaviour was reduced when medaka were presented with virtual medaka in which two features had changed. Fig 2B depicts the results of experiment 2. Time spent near the display was increased (at least transiently) in all groups when presented with visual stimuli. However, this trend was most prominent in the Normal group. As with experiment 1, the degrees of freedom were corrected because the sphericity assumption was not satisfied (*p* < 0.001). A two-way ANOVA indicated significant main effects of group (*F*(5,138) = 4.81, *p* < 0.001) and time (*F*(4,551.83) = 48.70, *p* < 0.001) and a significant interaction effect between group and time (*F*(19.99,551.83) = 1.72, *p* < 0.05). Over the entire duration of stimulus presentation, the time spent in proximity to the display in the Normal group was significantly longer compared with the baseline period (*p* < 0.05). Although the time spent near the display was also prolonged in other groups when compared with the baseline, we found the following significant differences at 1–5-min time points of the stimulus presentation period (all *p* < 0.05): at the 1-min time point, the Normal group vs. all other groups; at the 2-min time point, the Normal group vs. the Grey scale/No locomotion and Pressed/No locomotion groups; at the 3-min time point, the Normal group vs. the Grey scale/No locomotion group; at the 4-min time point, the Normal group vs. the Grey scale/No locomotion, Pressed/No locomotion, and Pressed/No body motion groups; and at the 5-min time point, the Normal group vs. all other groups. Visual stimuli used in experiment 2 were not as effective as the normal virtual medaka at inducing social approach behaviour.

### The effects of changing a single visual feature on social approach behaviours

The results of experiment 3 are represented in Fig 2C. As with experiments 1 and 2, the increase in time spent near the display was most evident in the Normal group. The degrees of freedom were corrected because the sphericity assumption was not satisfied (*p* < 0.001). A two-way ANOVA indicated significant main effects of group (*F*(4,115) = 4.60, *p* < 0.01) and time (*F*(4.05,465.73) = 39.52, *p* < 0.001). *Post-hoc* test revealed that the Normal group spent significantly more time near the display than the other groups (*p* < 0.05), with the exception of the No body motion group. Exposure to visual stimuli significantly prolonged the amount of time spent near the display (*p* < 0.05). Deviations from the Normal group reduced the time spent in social approach behaviour, although the medaka were not so sensitive to a lack of body motion information. These results suggest that although the induction of social approach behaviour was highly dependent on colour, shape, and locomotion information, any manipulation of the Normal group reduced the time spent in social approach behaviour, which is in accordance with our previous findings that indicated that deviations from the normal control group inhibited the induction of social approach behaviour [33].

## Discussion

Our study showed that the presentation of virtual fish created by 3DCG animations could induce social approach behaviour and that medaka were sensitive to changes in visual cues of virtual fish. 3DCG animation technology has been used in other behavioural contexts, such as responses to aggressive and submissive signals in lizards [36] and contagious yawning in chimpanzees [37]. Although 3DCG animation technology has been applied to study fish behaviour, including mate choice in sticklebacks [38], zebrafish [39], swordtail fish [40], and sailfin mollies [41], the number of studies using this technology is limited especially in the context of social approach behaviour, i.e., shoaling behaviour. It appears that virtual fish created by 3DCG animation technology can be a useful tool to study social approach behaviour.

Many behavioural studies have demonstrated the body colour of fish to be an important factor affecting shoaling behaviour [42–45]. For example, specific colour was reported to be significantly preferred in zebrafish [42]; while yellow-coloured images elicited a strong preference, red-coloured images were not preferred. The authors of that study discussed that yellow was treated by zebrafish as a sign of health, while red was considered to represent heterogeneous fish. In golden shiners, a robotic fish with a bioinspired colour pattern (grey robot) was more attractive than a red robot [44]. Red phenotypic variants of golden shiners are not present in nature [46], and thus their own body colouration might be preferred by golden shiners. The association toward a given stimulus in medaka fish was also highly dependent on colour, though, in our experiments, the issue of colour was dealt with simply by comparing coloured and grey scale models. Complicated and continuous spectra of visible light in the real world cannot be reproduced by the PC display in principle. Furthermore, eight colour opsins including UV-sensitive opsins (λmax = UV 356 nm, 405 nm; blue 439 nm; green 452 nm, 492 nm, 516 nm; red 561 nm, 562 nm) have been described in medaka fish [47]. These are not comparable to the visual system of humans (λmax = blue 435 nm, green 534 nm and red 560 nm), to which the PC display is optimized. Thus, studies of the visual colour signals using computer-manipulated stimuli should be performed in a careful manner, though a tool to adjust RGB imagery to be perceptually more realistic to subject animals was developed [48]. Nevertheless, our data derived from the simple comparison suggested that social approach behaviour is affected by colour, at least, to a varying degrees.

A reduction in social approach behaviours was observed when the body shape of the virtual medaka fish was altered. A similar observation was previously reported in a study using computer animated zebrafish [42]. While the “fat” looking shape (compressed) did not elicit any differential response from zebrafish compared to the unaltered control image, the “long and narrow” looking (stretched) image was not preferred by the zebrafish. This specific avoidance of a stretched-shape was discussed in terms of predator avoidance because a predator species (needle fish) has an elongated body shape. This may not be the case in our study, because no fish similar to our flat-shaped virtual fish (pressed models) exist under the aquatic environment where the medaka fish live. However, it is possible that pressed models were perceived as an unknown heterogeneous species.

Medaka fish were found to spend more time near the PC display when presented with moving animations compared with any static visual stimuli. These results were again consistent with previous studies performed with zebrafish [49]. The current study performed advanced manipulation of motion information (locomotion vs. body motion) for the first time. As a result, locomotion information was a more attractive visual stimuli compared to body motion information. When we presented medaka fish with virtual fish in which two features were changed concurrently, deletion of locomotion was the most effective means of reducing social approach behaviour. It is known that medaka fish swim with characteristic trajectories when they exhibit sexual and territory behaviours [50]. Males perform a dance in which they circle females at the time of reproduction. When starting territory action, they circle each other with characteristic trajectories. In the present study, however, we constructed virtual medaka based on female tracking coordinate data. Although female behavioural analyses were conducted using several paradigms, such as open-field, mirror-biting, and social interaction [20], we still understand little about the detailed characteristics of female medaka motion. It is, however, likely that female medaka have distinctive motion patterns compared with other species, and if such motions include a characteristic trajectory, they might be important factors in perception of conspecific fish.

It should be noted that a lack of body motion information led to a reduction in social approach behaviour, although not significantly. One of the advantages of the current study was that the various features, such as colour, shape, and motion, could be manipulated systematically. The systematic study using the same platform showed that any manipulated features could induce the repression of social approach behaviours to a greater or lesser extent. The most attractive visual stimulus for real medaka fish was “the original” virtual medaka fish.

Many researchers studying the relationship between visual stimuli and particular behaviours have searched for elemental features that are specifically responsible for a particular behaviour. For example, body pattern features of the crown butterfly fish [51] and zebrafish [39] can be used for conspecific identification of mates. The shape of the tail fin affects behavioural response to conspecifics in swordtail fish [52] and bettas [53]. However, our data suggests that all the perceivable features play some role in inducing social approach behaviour and that any difference in visual cues from the normal group can affect the perception of conspecific fish. It can be assumed that medaka fish have internal representation of conspecific animals, and that any strangeness derived from the differences between the internal representation and encountered conspecifics leads to the perception of non-medaka fish.

In the study context of mate choice and shoaling, some studies have supported the importance of combining multiple features. Using computer animated stickleback, Künzler and Bakker reported that the redness of the male body was preferred by females, and when the redness was combined with proper motion (zig-zag motion), the combined features revealed more pronounced female preferences [54]. A computer animation study in the cichlid also showed that a combination of both motion and specific body colour increased female preference for males [55]. Using videotaped images and 2DCG, Neri showed that combinations of proper motion and morphological features were important for shoaling behaviours of zebrafish [49].

Biological motion as well as 3DCG animations is an effective tool to study the mechanisms of visual processing and social behaviour in fish. Biological motion depicts a moving creature by means of only a small number of isolated points [56]. Visual stimuli of biological motion are devoid of colour or shape information and only contain locomotion and body motion information. Regardless of the lack of colour and shape information, visual stimuli of conspecific biological motion attract medaka fish [33]. It was found that deviation from “the original” familiar motion inhibited the induction of social approach behaviour. It appears that the naturalness of motion contributes to the induction of social approach behaviour. Again, we propose that in response to strangeness evoked by any perceived difference from the internal representation, the medaka fish change their behaviour. The subtraction between the internal representation and perception could be occurred in individual features. As a fundamental function of the brain, detection of differences between the anticipated and real values has been proposed [57–64]. Many neurological data from humans and primates suggest that separate brain areas are responsible for morphological and motion perception [65, 66]. Although it is unclear whether different visual features, such as morphological and motion, are analysed separately in the brain of fish, motion-sensitive neurons were found in several fish species, such as zebrafish [67], trout [68], dogfish [69], and goldfish [70]. The behavioural results of the current and biological motion studies may reflect the functional separation of the brain. Neurophysiological studies of medaka fish are needed to further clarify these issues.

In humans, highly realistic human-looking robots and computer-animated characters tend to elicit negative feelings. This phenomenon is referred to as “uncanny valley” [71]. The uncanny valley phenomenon could be occurred not only in humans, but also in monkeys [72]. Unfortunately, in the present study, there were no evidence which made it reasonably arguable that the uncanny valley phenomenon occurred because all visual stimuli elicited a certain degree of social approach behaviour and we could not find aversive visual stimuli. However, the present system can be useful to create more various visual stimuli. Further experiments will be needed to address this issue.

Computer animation technology is a powerful new tool that is being used to help answer questions related to animal behaviour. However, despite the advances provided by computer animation technology, the use of animation in animal behavioural research has not been without limitations. For example, real-time interactions between real fish and virtual fish is currently lacking. Such interactions could be of benefit when studying social behaviours or interspecific interactions. However, there are only a few studies which have developed and used the interactive animation systems for animal behaviour research [73, 74]. In the future, advanced tracking technology based on computer vision will enable virtual fish to effectively respond in real-time to real fish. Theoretical models using computer animations have predicted prey-predator and social interactions of fish [32, 75]. The incorporation of theoretical models into a real-time system will help computer animations shed light on the evolution of animal behaviour.

## Methods

### Animals and housing conditions

Adult medaka (*Oryzias latipes*, orange-red variety) were purchased from a pet shop, Focus (Kumamoto, Japan). The length of the bodies of all medaka was almost the same (about 3 cm). The medaka were maintained in 23 L glass aquaria for at least 7 days prior to beginning the experiment. The stock populations (approximately 40 fish per aquarium) were kept in aerated and filtered water at 26 ± 1°C. The holding water was prepared by mixing deionized water and artificial sea salt (0.3 g/L; Tetra Marine Salt Pro; Tetra Japan, Tokyo, Japan). The lighting cycle was 14-h light and 10-h dark (light from 06:00 to 20:00). The animals were fed an artificial dry diet (Tetra Killifish Food; Tetra Japan, Tokyo, Japan) twice a day (at 09:00 and 17:00). After the study, they were transferred to retirement aquaria and maintained. All experiments were approved by the committee for Animal Experimentation at the National Institutes of Natural Sciences, Japan (approval number: 14A050, 15A001, and 16A002) and carried out in accordance with relevant guidelines and regulations.

### Stimulus production

Photographs (width 5232 × height 3488 pixels) of the left, right, front, and top sides of each three female medaka were taken using a digital camera (Nikon 1 J4; Nikon, Tokyo, Japan). Although previously mentioned, the reason for choosing females was due to the fact that while males were preferred less by other males, both sexes were attracted to females in another fish species, zebrafish [24]. Specular highlights and small scars were eliminated using image manipulation software (GIMP2, http://www.gimp.org/). With reference to the photographs, the three dimensional shape of the three medaka fish were modelled using 3D modelling and animation software (3ds Max). Three textures were made from the photographs of three medaka fish, and the textures were pasted onto the polygon models using the additional software (Blender). To generate grey scale images, the photographs were converted to grey scale using GIMP2. These images were used for generating grey-scale virtual medaka fish (grey scale models). To generate pressed virtual medaka fish (pressed models), in which the width of the body was tripled, and the height of the body was reduced to one-third of its original size, the original polygon models were transformed using Blender.

The motion of the virtual medaka were reconstructed from the tracking data obtained from the real medaka. The details of how to record and analyse the motion of medaka were described in our previous paper [33]. Briefly, the movements of medaka fish were recorded from the side and above of an aquarium using digital video cameras (Himawari GE60; Library, Tokyo, Japan). The video images (width 640 × height 480 pixels) were recorded at 60 frames per second (fps). A small number of points (six points in the current study) were assigned at equal distance along the trunk of the body and tracked automatically in each video frame using motion analyser software (Wriggle Tracker; Library, Tokyo, Japan). Using the Wriggle Tracker, the position data were smoothed by a 3-point moving average method. Three movement patterns composed of three-dimensional coordinate sequences (3600 video frames, 1 min each) were constructed based on the video recording of three medaka fish (Medaka 1 to 3). The tracking coordinate data were then converted into the BVH file format, which includes skeleton hierarchy information and the motions of skeletons in each frame. In the BVH files, Euler angles were used to describe the rotations of the joints. In the current study, we generated five unit vectors formed by adjacent points, and then calculated the Euler rotation angles of each vector on each frame. The vertical axis of the body of the fish was fixed to the global vertical axis of Blender. The BVH files were imported into Blender, and morphological and motion information were linked. All animations were rendered in AVI format (1920 × 1080 pixels, black background). These animations were then converted to WMV files using computer software, and five-minute animations were constructed by connecting five of the same one-minute animations. Sample movies used in this study are shown in S1 to S3 Movies.

### Stimuli presentation

WMV animation files were presented on a 28-inch LCD with a refresh rate of 60 Hz and resolution of 3840 × 2160 pixels (inter-pixel distance: 0.16 mm). Visual stimuli were controlled by Visual Basic Express 2010 running on a Windows PC. All stimuli were presented within an area of 960 × 540 pixels (153.6 × 86.4 mm^2^) located in the centre area of the display. The size of the animated fish image nearly matched that of the real medaka fish. In the case of static image presentation (Static groups), images of the first frame of the animations were used. The static images from side view were used, and therefore could be recognised as medaka fish. In experiment 1, no images were presented to act as a negative control (Blank group). In our previous study [33], medaka were presented with biological motion stimuli of a single conspecific, but not a shoal, and it could effectively induce social approach behaviour. Fernandes et al. reported that animated images of a single conspecific induced social approach behaviour in zebrafish, though the inducing effects of a single conspecific animation on social approach behaviour were relatively small when compared with those of a shoal one [76]. Thus, we presented a single, but not a shoal, medaka fish animation.

### Behavioural test

Behavioural test was performed essentially as described in our previous paper [33]. Cubic glass aquaria (inner side length of 15 cm) were used as test tanks for the behavioural test. To restrict the exterior visual stimulation, the lateral sides were covered with white polystyrene sheets. The test tanks were filled with housing water (water depth: 8.0 cm). The tank water was maintained at a temperature of 26 ± 1°C by air conditioning.

The behavioural test consisted of an acclimatization period (23 h + 1 h), baseline period (1 min), and stimulus presentation period (5 min). A naïve fish was randomly selected from the stock populations and individually transferred to the test tank (from 09:00 to 17:00). Twenty-three hours later, the test tank was moved into the test room and attached to the LCD, and the animal was allowed to acclimatize for 1 h. In our previous study [33], abnormal behavioural responses were not elicited by separation for about one day from group members. The illumination at the surface of the water was adjusted to 2500 lx with two white florescent lamps. After the acclimatization period (23h + 1h), the behaviour of the animal was recorded continuously for 6 min from the side and above of the tank using the digital video cameras. The videos were recorded at a frame rate of 30 fps. The first 1 min was the baseline period, in which no stimuli were presented. Visual stimuli were presented on the LCD during the last 5 min (stimulus presentation period).

Behavioural analysis was conducted using self-built software (Medaka Fish Tracker, available at https://doi.org/10.6084/m9.figshare.4789045.v2). The coordinates of the tip of the fish’s head were automatically tracked in each video frame. The amount of time that the head was in close range to the LCD (an area of 10 mm in width from the inner surface of the tank on the display side) was calculated. Social approach behaviour was assessed based on the amount of time spent near the display. However, we did not use the simple mean value because subjects that incidentally tended to spend a longer time near the display were regarded as having higher social approach tendencies. We then excluded the medaka that stayed close to the display (within 10 mm) for an excessive amount of time (over 55 sec) during the baseline period, and calculated the differences in time spent near the display between the baseline period and the period of stimulus presentation. These differences were then analysed statistically.

### Experiment 1

In experiment 1, we constructed virtual medaka in which all four features (colour, shape, locomotion, and body motion) were included and compared the effects of normal virtual medaka and several types of static virtual medaka. We conducted behavioural test using 112 subjects. However, four medaka were excluded based on the behavioural analysis during the baseline period, leaving 108 subjects. An equal number of subjects were assigned to each of six groups: Normal, Static, Grey scale/Static, Pressed/Static, Pressed/Grey scale/Static, or Blank (each *n* = 18). In each group, half of the subjects were male, and the other half were female. In all groups except for the Blank group, one-third of the subjects, i.e., six subjects, were exposed to one of three visual stimuli (Medaka models 1 to 3). Because there were no significant sex differences in responses to the virtual stimuli (*p* > 0.05), the data from the male and female subjects were pooled. As noted above, male medaka perform a dance to court females [50]. However, in the present study, we have rarely seen such a characteristic movement pattern (personal observations). It is likely that both male and female medaka were socially (not sexually) motivated to swim to the virtual medaka. In addition, there were also no significant differences in responses to three variations in stimuli, i.e., Medaka models 1 to 3 (*p* > 0.05). Therefore, we pooled the data from the three variations. In most of the previous studies, it appears that virtual fish was constructed based on only one animal. By using three variations of visual stimuli, we reduced the problem of pseudo-replication [77], though three may not be enough. In the following two experiments, half of the subjects were male, and the other half were female. In addition, one-third of the subjects were presented with one of three visual stimuli based on the data from Medaka models 1 to 3.

### Experiment 2

Experiment 2 examined the effects of removing two features from the virtual medaka. Eight medaka were excluded based on the amount of time spent in proximity to the display during the baseline period, leaving 144 subjects to be analysed. An equal number of subjects were assigned to each of six groups: Normal, Grey scale/No locomotion, Grey scale/No body motion, Pressed/Grey scale, Pressed/No locomotion, or Pressed/No body motion (each *n* = 24).

### Experiment 3

In experiment 3, we modified a single feature of the virtual medaka and examined the contribution of these features to social approach behaviour. Based on the amount of time spent near the display during the baseline period, six medaka were excluded from the results, leaving 120 subjects. An equal number of subjects were assigned to each of five groups: Normal, Pressed, Grey scale, No locomotion, or No body motion (each *n* = 24).

### Statistical analysis

Time spent near the display in the baseline period was analysed using a one-way analysis of variance (ANOVA). Behavioural change from the baseline period was assessed using a two-way ANOVA with time as a within-subject factor (baseline, 1, 2, 3, 4, and 5 min) and group as a between-subject factor (Normal, Static, Grey scale/Static, Pressed/Static, Pressed/Grey scale/Static, and Blank groups in experiment 1; Normal, Grey scale/No locomotion, Grey scale/No body motion, Pressed/Grey scale, Pressed/No locomotion, and Pressed/No body motion groups in experiment 2; Normal, Pressed, Grey scale, No locomotion, and No body motion groups in experiment 3). Mendoza’s multisample sphericity test was conducted to test whether the assumption of sphericity had been violated. The Huynh-Feldt correction was applied when the sphericity assumption was not met. If the interaction was found to be significant, the simple main effects were analysed. When necessary, Ryan’s method was used for *post-hoc* multiple comparisons. A probability level of *p* < 0.05 was adopted as the level of statistical significance. All data are expressed as the means ± SD.

## Supporting information

S1 Movie Sample movie of normal virtual medaka (Normal group). The original movie was presented in the lower right corner.
(MOV)

S2 Movie Sample movies which were used in experiment 2. There were the following groups: Grey scale/No locomotion, Grey scale/No body motion, Pressed/Grey scale, Pressed/No locomotion, and Pressed/No body motion.
(MOV)

S3 Movie Sample movies which were used in experiment 3. There were the following groups: Pressed, Grey scale, No locomotion, and No body motion.
(MOV)
